# Variations within Class-A β-Lactamase Physiochemical Properties Reflect Evolutionary and Environmental Patterns, but not Antibiotic Specificity

**DOI:** 10.1371/journal.pcbi.1003155

**Published:** 2013-07-18

**Authors:** Deeptak Verma, Donald J. Jacobs, Dennis R. Livesay

**Affiliations:** 1 Department of Bioinformatics and Genomics, University of North Carolina at Charlotte, Charlotte, North Carolina, United States of America; 2 Department of Physics and Optical Science, University of North Carolina at Charlotte, Charlotte, North Carolina, United States of America; University of Wisconsin-Madison, United States of America

## Abstract

The bacterial enzyme β-lactamase hydrolyzes the β-lactam ring of penicillin and chemically related antibiotics, rendering them ineffective. Due to rampant antibiotic overuse, the enzyme is evolving new resistance activities at an alarming rate. Related, the enzyme's global physiochemical properties exhibit various amounts of conservation and variability across the family. To that end, we characterize the extent of property conservation within twelve different class-A β-lactamases, and conclusively establish that the systematic variations therein parallel their evolutionary history. Large and systematic differences within electrostatic potential maps and pairwise residue-to-residue couplings are observed across the protein, which robustly reflect phylogenetic outgroups. Other properties are more conserved (such as residue p*K_a_* values, electrostatic networks, and backbone flexibility), yet they also have systematic variations that parallel the phylogeny in a statistically significant way. Similarly, the above properties also parallel the environmental condition of the bacteria they are from in a statistically significant way. However, it is interesting and surprising that the only one of the global properties (protein charge) parallels the functional specificity patterns; meaning antibiotic resistance activities are not significantly constraining the global physiochemical properties. Rather, extended spectrum activities can emerge from the background of nearly any set of electrostatic and dynamic properties.

## Author Summary

Comparison of protein sequences and structures sharing function has become a well-established bioinformatics paradigm, leading to countless discoveries related to protein family sequence/structure/function relationships. However, sequence and structure alone provide only crude physiochemical descriptions, thus stressing the need for more sophisticated analyses. In this work, we determine how much dynamical and electrostatic properties vary across the β-lactamase enzyme family. Our results indicate that some properties are mostly conserved across the family, whereas others vary significantly despite the fact that all share the same high-level β-lactamase activity. Despite global variance in some metrics, systematic differences are frequently observed between evolutionary outgroups, indicating that physiochemical properties are simultaneously conserved and variable. As such, these results underscore the richness within physiochemical properties across a protein family and provide insight into how the variations came about.

## Introduction

The bulk of our knowledge concerning protein family evolution has come from comparative analyses of the large body of sequence and/or structure data produced over the last five decades. While this data has been invaluable to our current understanding, sequence and static structural descriptions provide only a narrow glimpse into stability and functional mechanisms. Consequently, there has been a growing interest to include physiochemical and functional details into molecular-evolutionary analyses [1]–[3]. For a complete understanding of these relationships, both conservation and variation must be characterized. Since conservation of function is the ultimate evolutionary driving force [4], protein orthologs tend to be significantly more similar in function than paralogs, and this functional similarity holds true with increasing sequence divergence as well [5]. Frequently, conserved functional patterns can be explained by conserved physiochemical properties [6], [7]. The β-lactamase (BL) enzyme family provides an excellent mix of preserved and adaptable physiochemical properties that require evolutionary/functional relation interpretation. On the functional aspect, BL enzymes have a chemically diverse set of substrates. Moreover, many BL enzymes can act on the same substrate despite being from evolutionarily distinct outgroups, leading to questions related about the presence (or absence) of conserved mechanistic strategies.

Antibiotic resistance continues to outpace our ability to bring new antibiotic drugs to market [8], leading to substantive fears about our continued ability to combat bacterial infections that are currently relatively benign. Central to this growing global health concern is the bacterial enzyme BL, which is produced by some bacteria [9]. BL confers resistance to penicillin and related antibiotics by hydrolyzing their conserved 4-atom β-lactam moiety, thus destroying their antibiotic activity [10]. Bacteria of all species depend on a cross-linked peptidoglycan layer, which preserves cell shape and rigidity. This peptidoglycan layer is primarily composed of alternating β(1,4)-linked monosaccharides, specifically N-acetylglucosamine and N-acetylmuramic acid. The latter is modified by a pentapeptide that always ends with two D-alanine residues. Cross-linking of peptidoglycan units is catalyzed outside the cytoplasmic membrane by cell wall transpeptidase enzymes. In this cross-linking process, a peptide bond is formed between penultimate D-alanine on one chain and pimelic acid (in Gram-negative) or L-lysine (in Gram-positive) residue on the other. The terminal D-alanine is cleaved off after the linkage is formed with the penultimate residue. β-lactam antibiotics effectively inhibit bacterial transpeptidases, consequently they are often called penicillin binding proteins (PBP). By inhibiting cell wall synthesis, the bacteria become highly susceptible to cell lysis.

In response, bacteria have evolved BL enzymes to defend themselves against β-lactam antibiotics. BL has, in fact, evolved from the functional domain of PBP through the acquisition of the new hydrolase activity [11]. The BL enzyme family is broad and is characterized by varying degrees of antibiotic resistance activity. In fact, extended spectrum β-lactamases (ESBL) also confer resistance to cephalosporins, which had previously eluded BL hydrolysis [12], [13]. ESBLs are evolved from traditional BL genes, generally through mutations within the active site [14], [15], thus highlighting the critical importance of subtle differences within members of the BL family. To date, more than 470 BL enzymes have been identified and are typically classified into 4 classes (A to D) based on sequence similarity [16]. Bush *et al.* developed a classification scheme for BL proteins based on their functional characteristics [17]. Protein structures belonging to classes A, C and D have similar folds and all have a mechanism that involves a catalytic serine residue, whereas class B enzymes are zinc metalloenzymes that have a distinct fold. In this work we focus on the most clinically relevant class-A family.

Comparing a number of different electrostatic and dynamical global properties, we quantify the extent of conservation across the class-A BL family. Our dataset includes twelve structures, each originating from a different bacterial species. We show that – as expected – many of the global properties are qualitatively conserved (such as residue p*K_a_* values, electrostatic networks, and backbone flexibility). Additionally, the local active site Ω-loop that is important for substrate recognition and catalysis is consistently established to be marginally rigid. However, some properties visually show large variance, and all properties have quantitative differences to varying degrees. In order to understand the origin of this variation, we quantitatively compare the differences within each property against the evolutionary clustering established by the family's phylogeny. Our results clearly establish that the systematic differences parallel the evolutionary patterns in a statistically significant way. To the best of our knowledge, this report establishes the most comprehensive and statistically robust relationship between physiochemical properties and evolutionary patterns. Going further, we also demonstrate the physiochemical properties parallel in a statically significant way the environmental condition of the bacteria they come from, which is not surprising since environmental segregation is likely related to divergence. Finally, we compare the same set of property differences to antibiotic specificity patterns. With the exception of enzyme charge, no correlations to antibiotic specificity are found, indicating that there is not a simple correspondence between global physiochemical properties and antibiotic specificity. This latter point is particularly alarming because it stresses that new antibiotic resistance patterns can emerge from a large fraction of the known BL enzymes through relatively small changes that do not significantly alter the global properties. Taken together, these results explain the variation within class-A BL physiochemical properties, while simultaneously suggesting new avenues of research regarding the plasticity within antibiotic resistance patterns.

## Results/Discussion

### Conservation and variation within residue pK_a_ values

Due to their clinical significance, serine-based class-A β-lactamase proteins are one of the most widely characterized enzyme families. The catalytic mechanism involves acylation of residue Ser-70 at the active site. However, identification of the general base that activates this serine residue has always been a subject of controversy. As such, two distinct residues have been proposed. While one hypothesis suggests that this role is played by the conserved Glu-166 [18]–[21], the other proposes Lys-73 [22]–[24]. In support of the first hypothesis, crystallographic data and MD studies [21] have suggested the presence of a conserved bridging water molecule that might act as a relay molecule for the transfer of proton between Ser-70 and Glu-166. Based on other experimental studies involving Glu-166 mutation [24], [25], the second hypothesis proposes an unsymmetrical mechanism involving two different general bases, Lys-73 and Glu-166 that carry out acylation and deacylation respectively. Swaren *et. al.* [26] have argued that substrate binding raises the p*K_a_* of Lys-73, which contributes to lowering of energy barrier for Ser-70, highlighting the importance of Lys-73 in proton transfer. Conversely, kinetic studies of several Glu-166 mutant enzymes [27] have displayed decreased rates of acylation and deacylation, emphasizing that Glu-166 is more important. Due to this absence of Glu-166 negative charge in mutant proteins, the Lys-73 side chain exhibits a lower p*K_a_* shift, acting as an alternate general base in hydrolyzing β-lactam ring [28]. Regardless of which hypothesis is correct, the above studies clearly highlight the importance of both Lys-73 and Glu-166.

**Figure 1:**
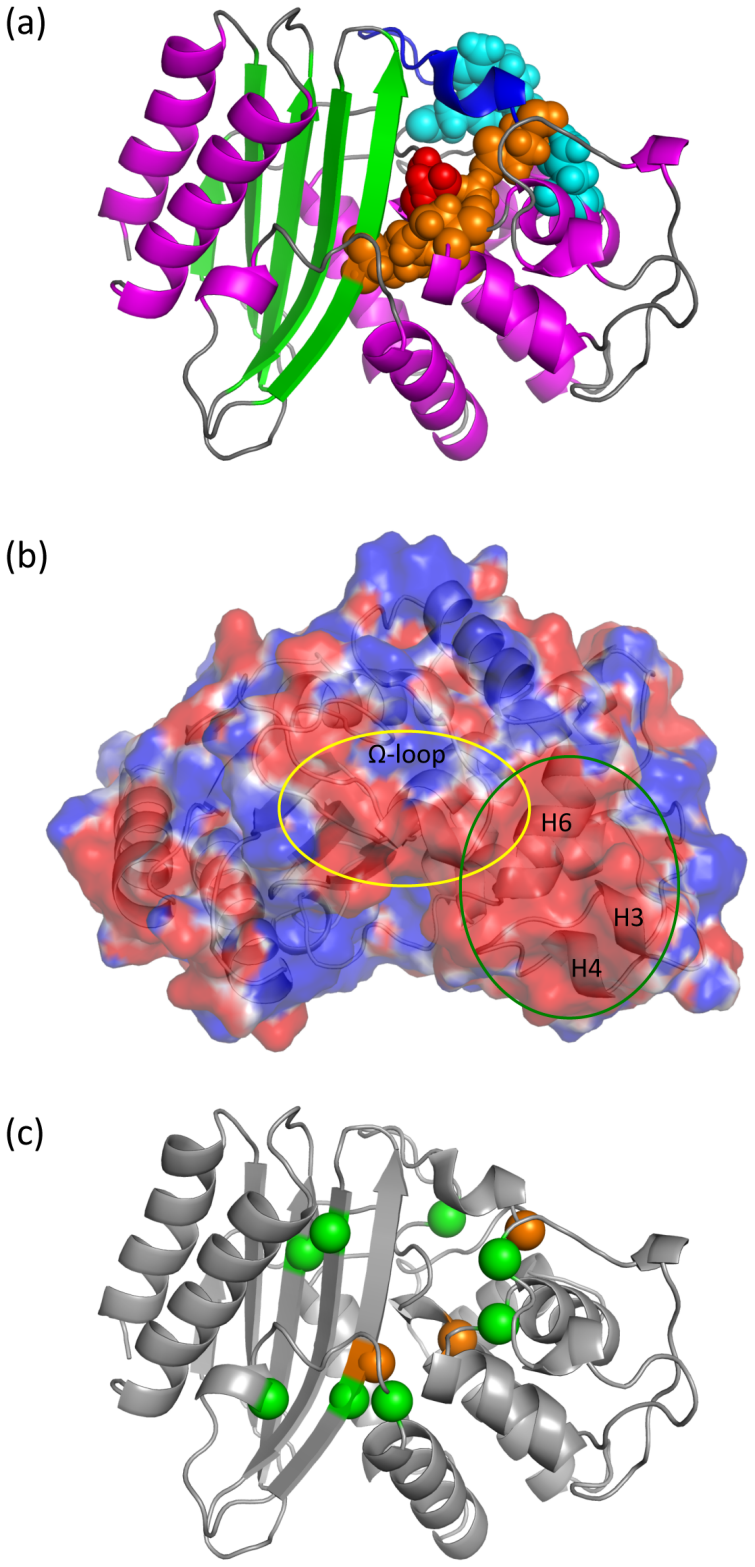
Significant β-lactamase residues and its electrostatic characterization. (a) Structure of a Class-A β-lactamase enzyme. The active site is located at the domain interface. The catalytic residue Ser-70 is shown in red. Other catalytic residues are shown in orange, whereas the Ω-loop is shown in blue at the top. Residues that maintain the structural integrity are shown in cyan. (b) Electrostatic potential values of ±1 kcal/mol are mapped to the protein surface. Red indicates negative potential, while blue indicates positive potential. The structure is oriented to display a patch of negative potential at the interface of the Ω-loop and helices H3, H4 and H6 that is conserved within the TEM/SHV enzymes. (c) Conserved electrostatic networks (cf. **Figure 2b**) are mapped to a BL structure. Green colored spheres represent α-carbons of residues interacting strongly with catalytic residues, which are highlighted in orange.

Several other residues have also been identified in BL that are catalytically important: Ser-70 being the primary catalytic residue; Lys-73, Glu-166, Ser-130, Lys-234 as secondary catalytic residues. Finally, Asn-136, Arg-164, Asp-179 are other important residues that maintain the active site structure (Figure 1a). All of these residues are in spatial vicinity of Ser-70 and affect substrate recognition and catalysis. Detailed sequence and structural comparison across the class-A family has identified similar structural and functional elements that span over active site residues mentioned above [29]–[32]. These conserved elements are SxxK, SDN, ExxLN and KTG.

**Figure 2:**
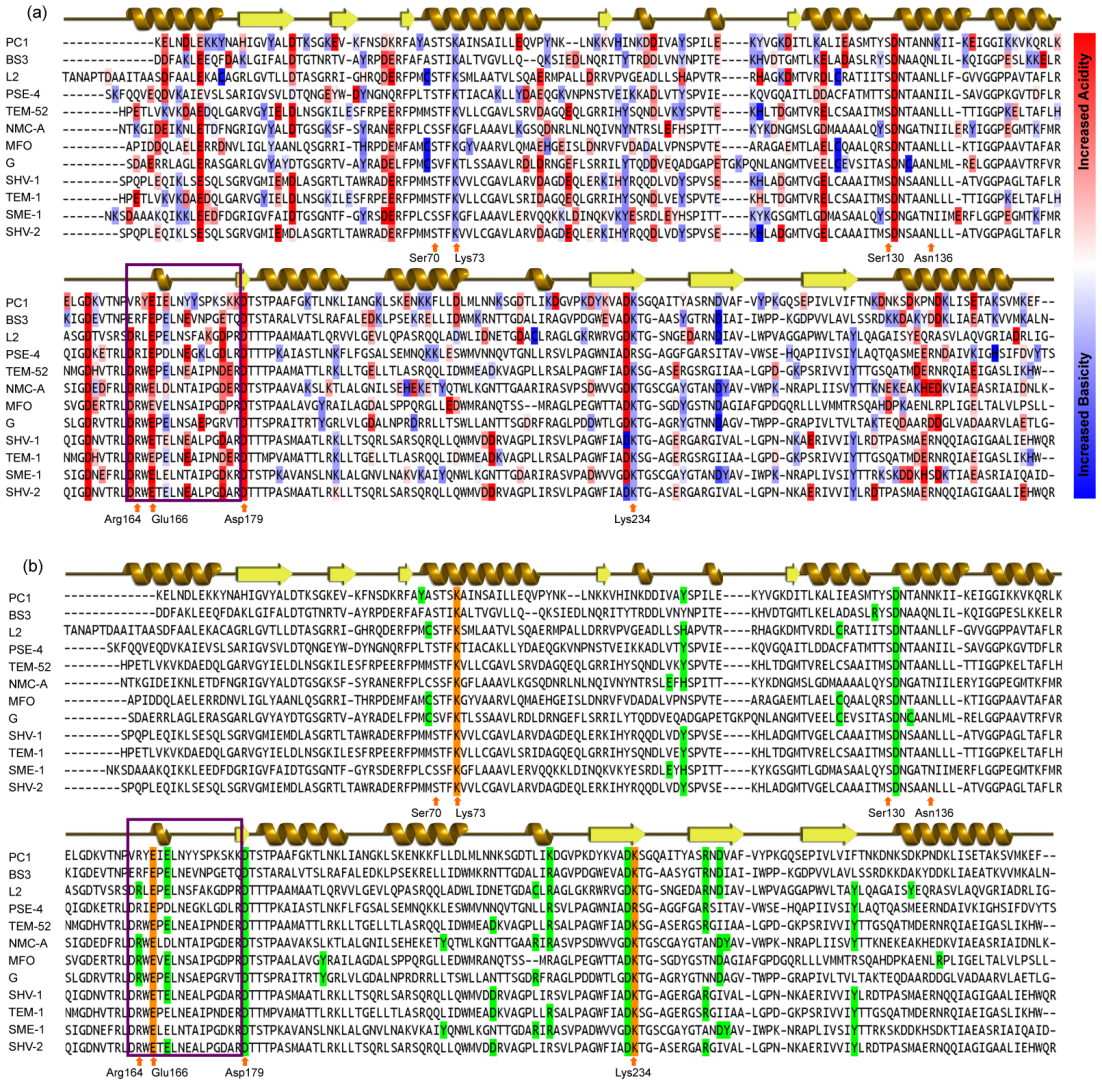
Electrostatic properties of β-lactamase family. (a) Multiple sequence alignment of twelve β-lactamases color-coded by shifts in residue p*K_a_* values from model values. Residues colored red express increased acidity, whereas residues colored blue show increased basicity. (b) Residues colored green exhibit strong (>|±1| kcal/mol) electrostatic interactions with catalytic residues that colored orange. The identified residues are also highlighted in the β-lactamase structure provided in **Figure 1c**. The Ω-loop region is indicated by the purple box. A cartoon representation of secondary structure is displayed on top of each alignment, while active sites are displayed below.

Conservation of important electrostatic properties is a commonly employed mechanism that leads to conserved function [6], [7]. Figure 2a shows calculated residue p*K_a_* shifts (shifted away from their model values) across twelve BL proteins. Interestingly, these p*K_a_* shifts are mostly conserved, emphasizing a common mechanistic strategy. We further investigate the site-site interactions of residues that have strong electrostatic interactions (more than 1 kcal/mol) with the secondary catalytic residues Lys-73, Glu-166 and Lys-234 (Figure 1c). Remarkably, all conserved electrostatic sites overlap with the four conserved element regions, highlighting the strength of the active site electrostatic forces. All pairwise active site interaction energies are listed in Table 1. Further, all these sites have a conserved p*K_a_* shift. Asp-131, Glu-166, Asp-179 and Asp-233 display strong acidic character, whereas Lys-73 and Lys-234 exhibit conserved basic shift in p*K_a_*. Lys-73, which acts as proton extractor from Ser-70, needs to be deprotonated for acylation. As such, there is a cationic electrostatic microenvironment surrounding Lys-73, which is created by nearby basic residues Lys-234 and Arg-244 [24]. When Arg-244 is missing (which is the case in the NMC-A, MFO and G orthologs), this role is acquired by Arg-164 as shown in our active site electrostatic networks plot (Figure 2b). Another important feature of BL proteins is the Ω-loop (comprising of residues 163–178) that is involved in substrate recognition. Additionally, the Ω-loop comprises Glu-166, which is critical for deacylation activity. Our results reveal a strongly conserved acidic behavior within Glu-166, which activates a water molecule in the vicinity to attack carbonyl carbon of the acyl-enzyme. This ensures a back-delivery of the abstracted proton to Ser-70 γ-O atom, leading to enzyme regeneration [21].

**Table 1:** Summary of the active site electrostatic network^1^.

	Lys-73	Asp-131	Glu-166	Asp-179	Asp-233	Lys-234	Ser-70
**Lys-73**	–	6.0 (8.5)	2.8 (10.2)	10.9 (5.4)	11.2 (4.8)	4.1 (8.3)	3.1 (12.8)
**Asp-131**	−3.4 (17.7)	–	8.1 (5.1)	16.7 (2.6)	14.6 (4.9)	8.3 (4.9)	8.7 (6.7)
**Glu-166**	−7.6 (15.3)	1.7 (12.0)	–	8.1 (5.6)	15.0 (4.2)	7.7 (7.2)	3.4 (27.9)
**Asp-179**	−1.0 (11.8)	0.3 (8.6)	1.7 (9.6)	–	19.3 (3.0)	13.9 (4.7)	10.2 (4.5)
**Asp-233**	−0.9 (10.4)	0.4 (11.2)	0.5 (8.0)	0.3 (10.9)	–	5.3 (4.2)	11.3 (8.4)
**Lys-234**	4.0 (13.0)	−1.5 (9.5)	−1.7 (9.6)	−0.6 (13.9)	−3.3 (12.8)	–	4.7 (9.6)

1 With the exception of the last column, values in the upper triangle provide the minimal distance (in Å) between atom pairs in the two residue side chains, whereas values in the lower triangle quantify the pairwise electrostatic potential energy (expressed in kcal/mol).

Values in the last column provide the minimal distance (in Å) between atom pairs of the electrostatic network residues and the catalytic Ser-70. The reported values are the average across the dataset, and the coefficient of variation is shown in the parentheticals (expressed as a percent).

### Conservation and variation within electrostatic potential maps and protein charge

**Figure 3:**
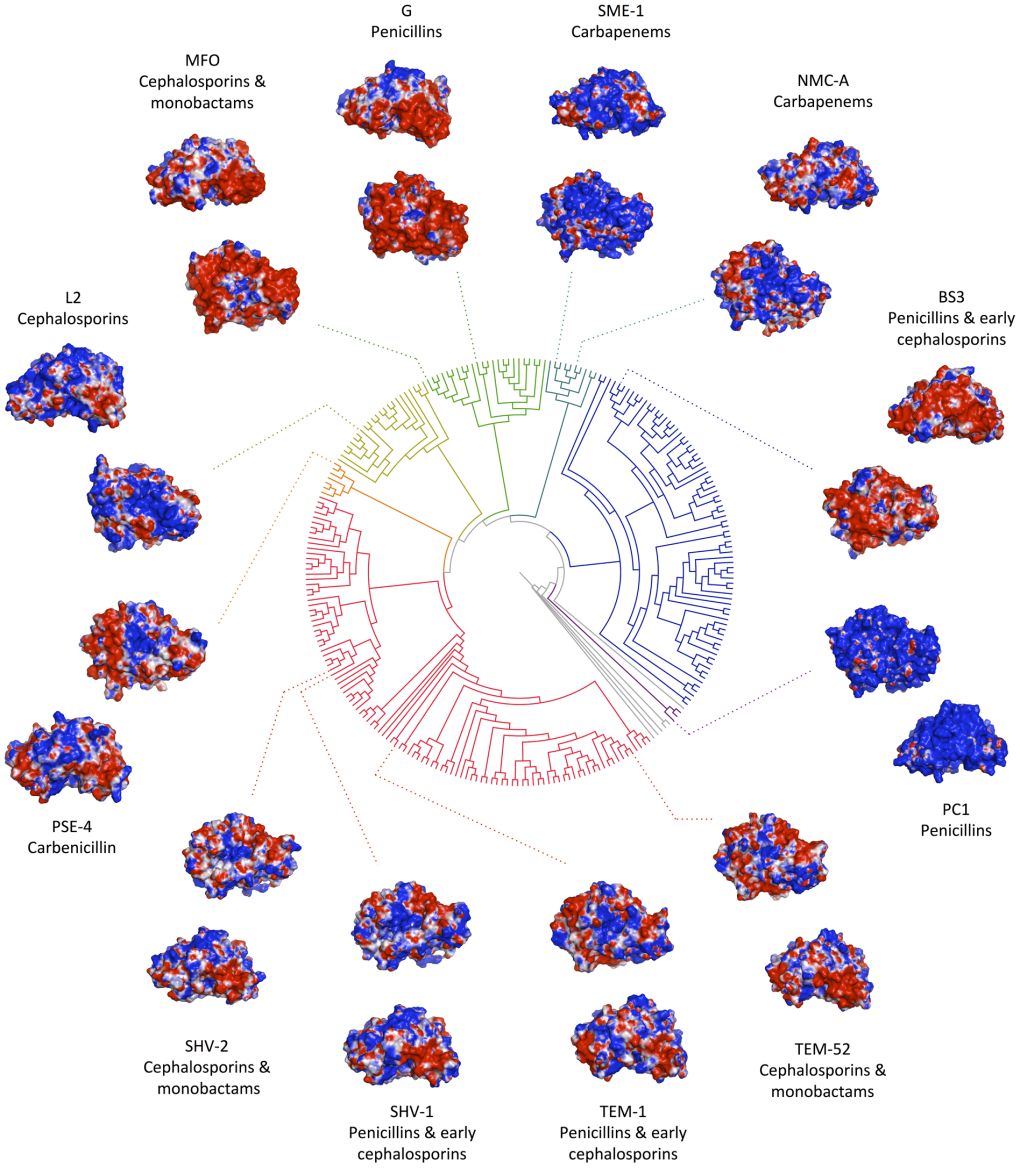
Relationship between phylogeny and electrostatic potential maps. The class-A β-lactamase family phylogeny is shown, which differentiates into 7 subgroups using a constant cut-level. Outgroups are represented by a unique color for better visualization. The structures closest to the phylogeny are oriented to highlight the active site region, which is indicated in green in the TEM-1 ortholog. Structures in the outer ring have been rotated in the y-direction by approximately 90 degrees, which highlights the Ω-loop region, also indicated in green. It is clear that structures from the same outgroup have visually similar electrostatic potential maps, whereas there are significant differences across the whole phylogeny.

The above results highlight the importance of conserved local electrostatic properties, whereas Figure 3 demonstrates that global electrostatic potential maps can be quite varied across the whole family. For example, the BS3 structure is primarily anionic, whereas the PC1 penicillinase is mostly cationic. Nevertheless, key features within the electrostatic potential maps are visually conserved within evolutionary outgroups. This point is exemplified by the TEM/SHV enzymes that have a conserved anionic patch spanning helices H3, H4, H6 and the Ω-loop (cf. Figure 1b); however, the patch typically missing from structures outside this outgroup. Similarly, other outgroups conserve visual electrostatic features, yet no potential map features visually align with antibiotic activity patterns.

Differences within the electrostatic potential maps are not unexpected owing to the sequence and structural variability within the dataset. Pairwise sequence identities range from 27% to 98%, which translates to α-carbon root mean square differences up to 2.6 Å. Moreover, the net charge of these twelve enzymes ranges from −6 to +15 (Table 2). This large structural variation with distinct electrostatic properties raises the question, “How does nature maintain the common functionality of enzymes?” Key sequence/structure motifs provide an insight into the description of the underlying conservation. Sequence conserved regions SDN and KTG have a strictly conserved charge of −1 and +1, respectively, across all twelve BL enzymes. Interestingly, the other two key regions, SxxK and ExxLN, which have variable sites x, are also strictly conserved with a charge of +1 and −2, respectively. ExxLN lies within the 16-residue Ω-loop (xRxExxLNxxxxxxxx) that maintains an overall negative charge (except PC1) ranging from −2 to −4. The conserved electrostatic properties of key regions range from simple local conservation of charge to complex evolutionary origins of BLs. Conservation of charges at mutable motifs and Ω-loop are achieved through concerted mutations. When there is a charge changing mutation at these important electrostatic regions, there is a charge compensating mutation elsewhere.

**Table 2:** Characterizations of charge and H-bond properties.

Enzyme	SxxK Charge	ExxLN Charge	Ω-loop Charge	Total Charge	Total HB Energy	Number of HB	Avg. HB Energy
G	1	−2	−2	−3	−1631.3	559	−2.9
TEM-1	1	−2	−4	−5	−1609.6	513	−3.1
NMC-A	1	−2	−3	1	−1588.3	520	−3.1
SME-1	1	−2	−1	7	−1728.4	550	−3.1
PSE-4	1	−2	−2	−4	−1548.0	529	−2.9
TEM-52	1	−2	−4	1	−1590.9	557	−2.9
L2	1	−2	−1	2	−1534.6	528	−2.9
SHV-2	1	−2	−3	1	−1633.2	539	−3.0
SHV-1	1	−2	−3	1	−1570.7	532	−3.0
MFO	1	−2	−2	−6	−1503.0	507	−3.0
PC1	1	−2	2	15	−1559.3	495	−3.2
BS3	1	−2	−4	−5	−1669.1	522	−3.2
**Average**	**1**	**−2**	**−2.3**	**0.4**	**−1597.2**	**529.3**	**−3.0**
**CV** 1	**0.0%**	**0.0%**	**76.1%**	**1430.7%**	**3.9%**	**3.7%**	**3.8%**

1 Coefficient of variation = standard deviation/mean * 100.

### Differences within electrostatic properties reflect evolutionary patterns

The preceding sections reveal a rich mixture of conservation and variability within p*K_a_* values of important residues, charge, and electrostatic potential maps. Moreover, even when properties are visually conserved, quantitative differences are almost always present. Can the propagation of these differences across the family be explained? To answer that question, we perform statistical tests (cf. Methods) to elucidate the hidden relationships between enzyme sequence and physiochemical properties. Specifically, we test the statistical significance of pattern relationships between the global properties and the evolutionary outgroups defined from the BL phylogeny. That is, are differences within these global properties suppressed within phylogenetic outgroups relative to differences across multiple outgroups? The answer is yes, we indeed find this to be the case for all electrostatic properties, including electrostatic network pairwise energies, electrostatic network composition, residue charge, and per residue p*K_a_* shifts (cf. Table 3). As such, these results conclusively establish that the observed variations within physiochemical properties, which can at times be extreme, are robustly defined by the phylogeny, thus indicating that variation within these global physiochemical properties is an evolutionary driving force underlying BL divergence. Conversely, variations observed with the local active site Ω-loop do not reflect the phylogenetic clustering because these properties are too conserved based on strict mechanistic requirements imposed on all BL enzymes.

**Table 3:** P-values from the statistical z-test comparing physiochemical patterns to two different clustering sets.

Property^1^	Evolutionary Relationship	Environmental Condition	Antibiotic Specificity
H-bond contact map	^**^1.2×10^−3^	^**^5.3×10^−3^	2.2×10^−1^
Residue charge	^**^3.6×10^−3^	^**^1.3×10^−2^	^**^1.4×10^−2^
Electrostatic interaction network energy	^**^3.9×10^−3^	^**^1.4×10^−2^	8.3×10^−2^
Electrostatic interaction network composition (RM)	^**^4.8×10^−3^	^**^9.4×10^−3^	1.0×10^−1^
H-bond density per residue	^**^6.2×10^−3^	^**^4.4×10^−2^	3.3×10^−1^
Change in p*K_a_*	^**^8.4×10^−3^	^**^2.4×10^−3^	8.2×10^−2^
Cooperativity Correlation (CC)	^**^1.8×10^−2^	5.3×10^−2^	4.8×10^−1^
Backbone Flexibility (FI)	^**^1.8×10^−2^	^**^2.7×10^−2^	2.4×10^−1^
Ω-loop residue charges	8.2×10^−2^	2.3×10^−1^	^**^1.9×10^−2^
Ω-loop delta p*K_a_*	8.3×10^−2^	2.3×10^−1^	8.9×10^−2^
Ω-loop FI	1.7×10^−1^	2.1×10^−1^	1.4×10^−1^

1 Values less than 0.05 (indicated by **) signify structure/function/evolutionary relationships.

Similarly, we divided the BL family into four groups based on the environmental conditions of the bacteria they are from. First, we stratified the dataset based on whether the bacteria is aerobic or facultative anaerobic, and we additionally stratified based on whether the bacterium is gram-positive or gram-negative, which affects the locations of where the bacteria are likely to be found. For example, gram-positive bacteria tend to survive in dry conditions and are found in places like skin or in dust, whereas gram-negative bacteria thrive in aqueous conditions. As before with phylogenetic outgroups, we find that the variations within the electrostatic network pairwise energies, electrostatic network composition, residue charge, and per residue p*K_a_* shifts reflect environmental condition in a statistically significant way. Through congruence, the two sets of results clearly indicate that environmental condition has played an important role in BL evolution. This is not new [33] or surprising, but it does represent the first time that physiochemical properties were used to demonstrate the relationship between the two.

One of the most attractive features of the BL system is that, in addition to the phylogeny, the family can also be clustered based on antibiotic specificity. Performing the same analyses a third time, but now based on the antibiotic specificity patterns, only one (charge) of the electrostatic properties reflects the physiochemical properties in a statistically meaningful way. This indicates that antibiotic specificity patterns are not confined to narrow property ranges, and that the considered properties do not drive the global divergence of the family. This result is interesting and surprising considering the common view that function is the ultimate evolutionary driving force. Moreover, from a public health point of view, this result is alarming because it highlights that new activities can emerge from any global property background. Put otherwise, new antibiotic resistance activities, including those found in ESBLs, are evolutionary easy to achieve because they come about through small changes that do not globally affect structure and the concomitant electrostatic properties (electrostatic network pairwise energies, electrostatic network composition, residue charge, and per residue p*K_a_* shifts).

The only physiochemical property that reflects the functional patterns in a statistically significant way is residue charge at pH = 7.0, which is consistent with several prior works demonstrating the importance of charge-charge interactions within BL function and specificity. For example, Selzer et al [34] designed new BL proteins by altering surface charged residues that increase association rates. This change in biophysical property leads to changes in long-range electrostatic forces that may even change its functional specificity. Formation and breaking of ionic interactions in directed evolution experiments have also been exploited to design new proteins with distinct substrate activities [35], [36].

### Conservation and variation in flexibility/rigidity properties

**Figure 4:**
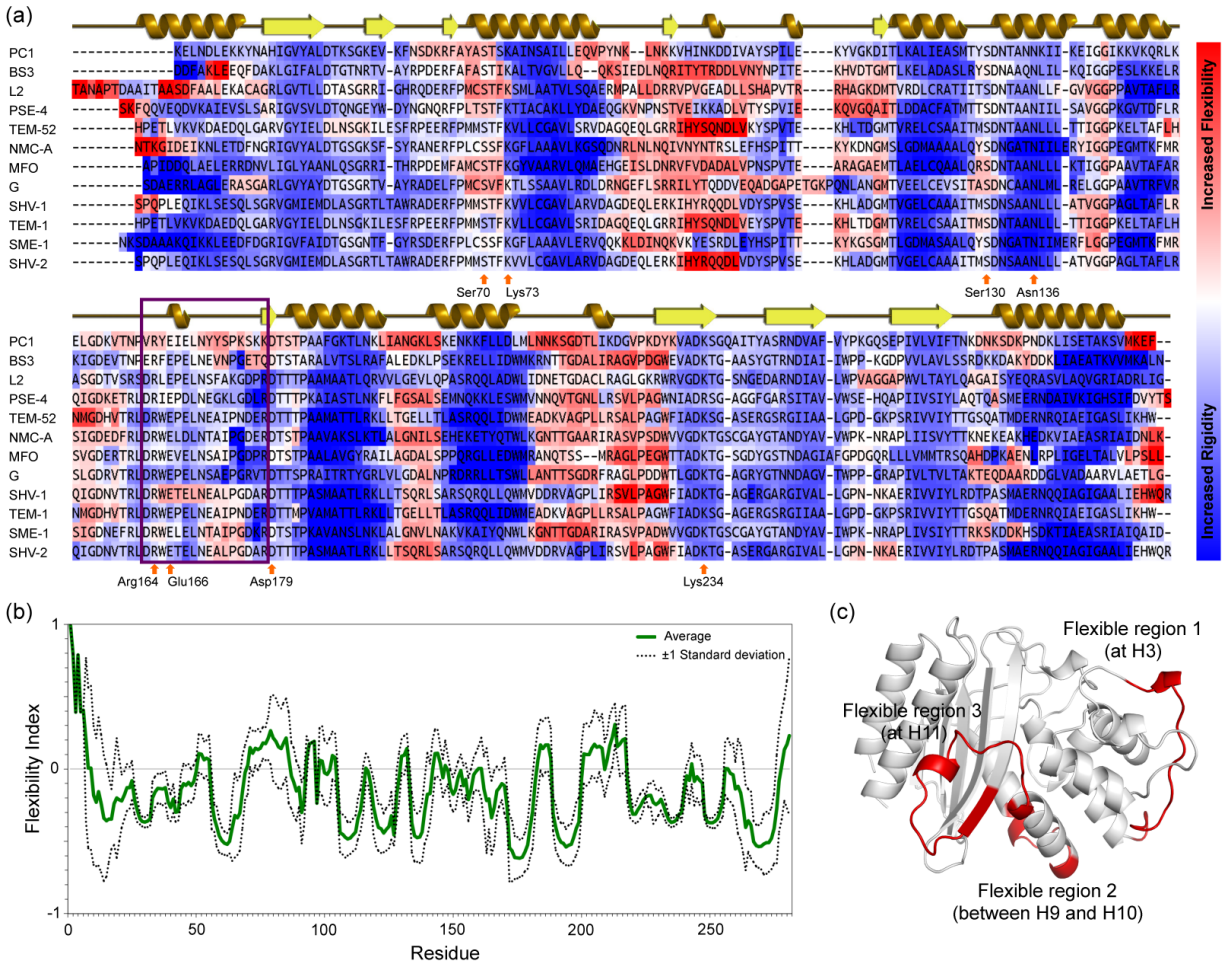
Backbone flexibility of β-lactamases is well conserved. (a) The flexibility for each structure is mapped onto the multiple sequence alignment of the class-A β-lactamase family. The backbone of residues colored red is flexible, whereas blue indicates rigidity. The spectrum bar illustrates the extent of flexibility and rigidity, which ranges from −1 to +1. (b) Flexibility index values averaged across the family are shown in in green, whereas the dashed lines highlight fluctuations (as defined by ±1 standard deviation). (c) Visual observation of backbone flexibility identifies three main flexibility regions that are mapped on to the structure. These flexible loops might have an important role in protein functionality.

Using the minimal Distance Constraint Model (mDCM), which we have used to characterize dynamic properties across several different groups of related proteins [37]–[42], we also characterize the extent of dynamical changes in BL. This work is particularly important because only a small number of class-A BL proteins have been studied by NMR and molecular dynamics simulation. As such, little is known about variation and conservation of dynamical properties across the BL protein family. Figure 4a displays the multiple sequence alignment of twelve BL proteins color-coded by flexibility index (FI), which quantifies local flexibility along the protein backbone. Residues colored blue are rigid, whereas the ones colored red are flexible. Figure 4b quantifies the average FI across the complete dataset displaying average FI curve with +/−1 standard deviation. Positive FI values reflect the amount of excess degrees of freedom in flexible regions, and negative values reflect the amount of excess constraints in rigid regions. These results highlight two significant points. First, BL enzymes have a predominantly rigid backbone, and second, this backbone rigidity is conserved across the whole family. Normally, our calculations do not predict structures to be so rigid, but this prediction is consistent with NMR S2 order parameter descriptions [43]. The extent of rigidity is also visible at the N and C termini of BS3, TEM-1, SME-1 and SHV-2. The flexibility/rigidity results of BL proteins presented in Figure 4a are rank ordered based on increasing average rigidity characteristics.

Across the alignment, the secondary structure elements appear rigid, whereas intervening loops are flexible (except the Ω-loop). Three flexible regions have been identified as shown in Figure 4c: flexible region 1 at helix H3, flexible region 2 between H9 and H10 and finally flexible region 3 at H11. While helix H10 is rigid, it is sandwiched between two flexible regions, meaning it could also have high mobility because the rigid body can “swing” from the flexible hinge in the same way a pendulum swings at a flexible pivot. We point this out because molecular dynamic studies have shown increased mobility in helix H10 upon substrate binding [44].

**Figure 5:**
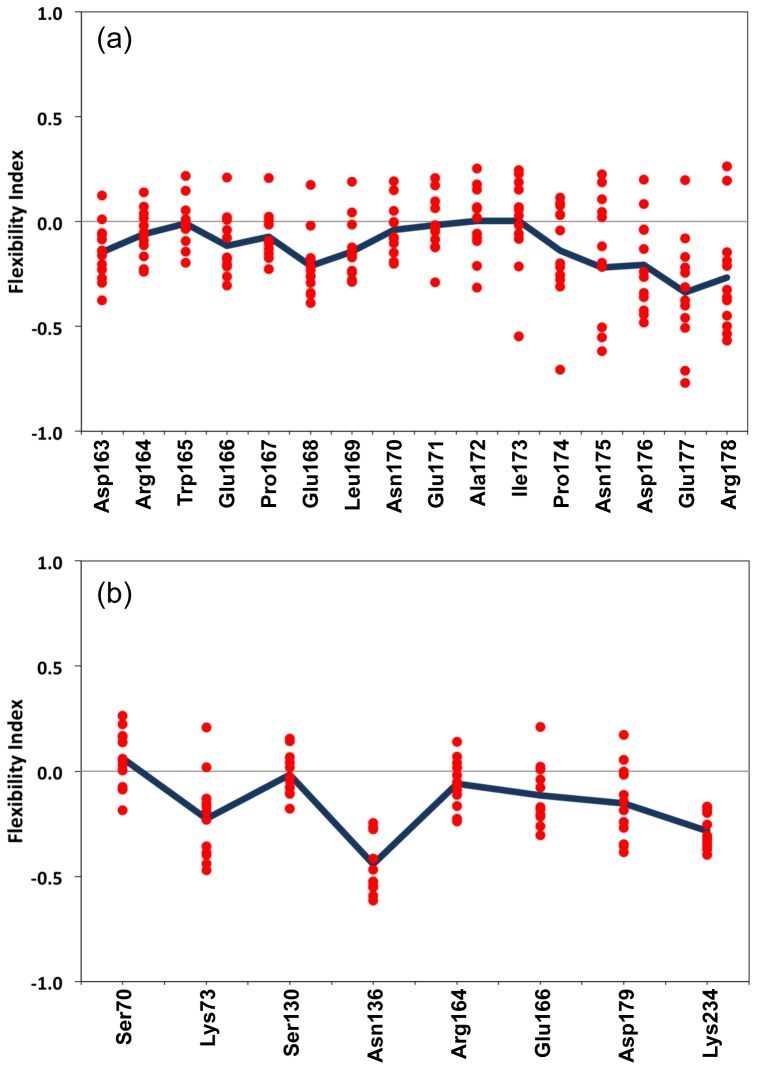
The backbone flexibility index reveals the nearly conserved isostatic nature of both (a) the Ω-loop and (b) the eight active site residues. The black line indicates the average value. Marginal rigidity is able to maintain the active site structure, while also allowing for the flexibility needed for substrate recognition and catalysis.

Mobility within the Ω-loop is thought to be important for substrate recognition and catalysis. Dynamic simulations performed in the past have suggested that the Ω-loop is rigid with order parameters comparable to other secondary structure elements [45]. The authors also illustrate the importance of flexibility at the tip of the Ω-loop, which is important for the opening and closing motion. Interestingly, mDCM results indicate that the Ω-loop is consistently isostatic, that is, marginally rigid along with eight active site residues (cf. Figure 5). As discussed above, the Ω-loop includes a key catalytic residue, Glu-166, that performs the deacylation step. Furthermore, deletion of the Ω-loop makes the protein deacylation deficient resulting in the formation of stable acyl-enzyme complexes [46]. The marginally rigid Ω-loop suggests its catalytic importance where rigidity is important for reproducibility in substrate binding, yet also allowing for motion that might be functionally required. The Ω-loop region spans over three out of eight catalytic residues. Except Asn-136, all catalytic residues exhibit similar isostatic nature even though they occur throughout the BL sequence.

In stark contrast to the global variability observed across the BL dataset, the marginal rigidity and electrostatic properties of the active site region are conserved. In most cases, small increases in new activities can be directly attributed to only a handful of active site mutations that sterically allow new substrates to bind [10]; yet active site rigidity is maintained. In fact, this active site rigidity was recently utilized to develop new BL inhibitors using a fragment based drug design strategy [47]. These results support the view that steric and electrostatic complementarity between active site and different antibiotics are primarily responsible for BL resistance activities [48]. Note however that the CTX-M BL enzymes do show increased active site flexibility [49], while maintaining active site geometrics consistent with the narrow spectrum TEM-1 and SHV-1 enzymes, thereby stressing their mechanistic plasticity within antibiotic resistance activities. Note that the CTX-M structures do not meet our structural quality criteria (cf. Methods); as such, they are not included in these analyses.

**Figure 6:**
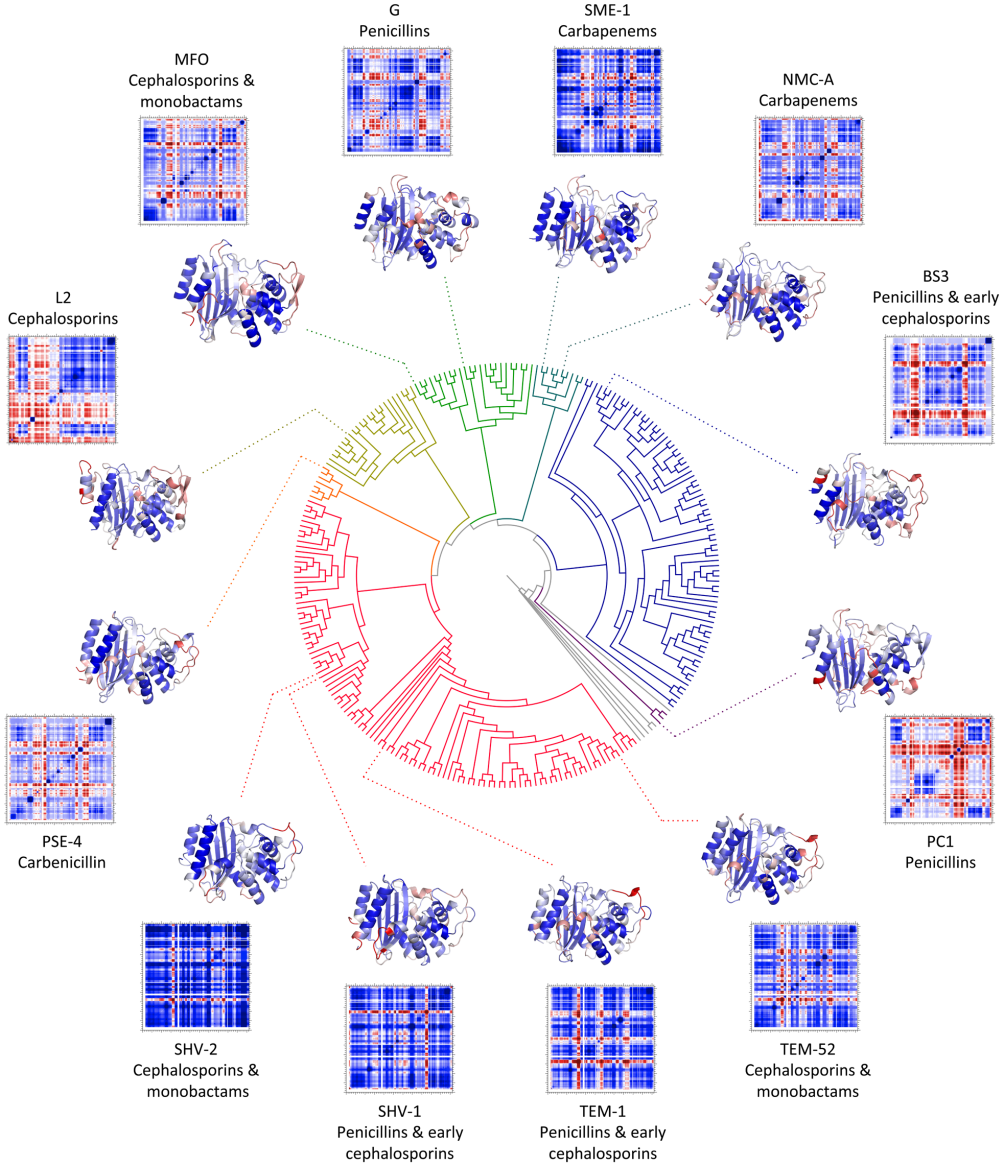
The phylogenetic tree along with the corresponding protein structures and cooperativity correlation plots. Sequence and structure dynamics are evolutionary related as evident from cooperativity correlation clustering. Structures are color coded by backbone flexibility index, which illustrates that all β-lactamase family members are primarily rigid with some punctuating flexible loops. Conversely, pairwise allosteric couplings are overall varied, yet typically conserved within evolutionary outgroups.

In addition to backbone flexibility, the model also calculates a correlation metric called cooperativity correlation (CC) that describe pairwise mechanical couplings. As illustrated in Figure 6, CC between a pair of residues in the native state can be rigidly correlated (colored blue), flexibly correlated (colored red), or uncorrelated (colored white). Taken together, the full CC plot can help elucidate allosteric couplings within structure. In a previous investigation of periplasmic binding proteins [38], the variability within the cooperativity correlation was explained by differences within the H-bond network. Interestingly, the H-bond network of BL proteins remains conserved (discussed below), yet we observe substantial diversity and richness of CC throughout our dataset. In this way, the results presented here are much closer to our results with thioredoxin [40], CheY [50] and lysozyme [42] that stress the sensitivity of CC, and thus allostery, to subtle structural perturbations. To further investigate this susceptibility within BL, we again layer the physical descriptions of structure onto the BL phylogeny. As with the electrostatic potential maps, CC properties again cluster in a way that reflects local evolutionary outgroups (cf. Figure 6). For example, TEM-1, TEM-52, SHV-1 and SHV-2 are largely composed of a single rigid cluster, which is consistent with earlier NMR [43] and MD [51] assessments of TEM-1 that indicated it is quite rigid. Carbapenemases SME-1 and NMC-A represent a close evolutionary pair, and thus have similar flexibility properties. Conversely, the L2 cephalosporinase, which belongs to a distinct outgroup, is atypically flexible.

As before, the global FI and CC metrics also reflect the evolutionary and environmental condition patterns in a statistically significant way, but not the antibiotic specificities. Comparison of the two penicillinases within the dataset provides an illustrative example of how large the differences within the physiochemical properties can be, even among enzymes sharing antibiotic resistance activities. The backbone of penicillinase PC1 is the least rigid structure characterized, and it also has a very atypical cationic electrostatic surface. However, neither property is shared with penicillinase G. Its surface is primarily anionic and its backbone is significantly more rigid than the average structure. Significant CC differences between the pair are also observed. While large, the differences within the penicillinase pair are not outside ranges established by our whole dataset, especially considering the early evolutionary divergence between PC1 and G established by the phylogeny.

Multiple attempts to relate electrostatic and rigidity relationships were made, but all were unsuccessful. Nevertheless, these results clearly demonstrate how systematic differences within electrostatic properties and CC parallel the overall phylogeny across BL enzyme family. Further, it is interesting to note how nature preserves the active site dynamics and their electrostatics properties during evolution. Conservation of function provides the selection bias for proteins to maintain globally similar dynamics while evolving to varying substrate recognition patterns.

### Conservation and variation in hydrogen bond networks

Table 2 describes the global H-bond statistics showing the number of H-bonds and average total energy across the twelve BL structures. Since the mDCM is in large part based on H-bond networks, it is critical to understand how their variation can affect dynamical properties. H-bond statistics show that the number of H-bonds varies from 495 to 559, whereas the average H-bond energy ranges from −2.86 to −3.20 kcal/mol. In our previous studies we have noticed that the number of H-bonds can be trivially explained by the size of the protein [38]. However, due to their relatively constant size, no such correlation is observed here. We also find that the above variations do not trivially predict differences within backbone FI and CC. That is, structures with more H-bonds are not necessarily more rigid than those with fewer. As we have discussed previously [40], [42], this observation again stresses that topological considerations get lost in global metrics due to nonadditive nature of the mDCM, which has a considerable effect on the output.

**Figure 7:**
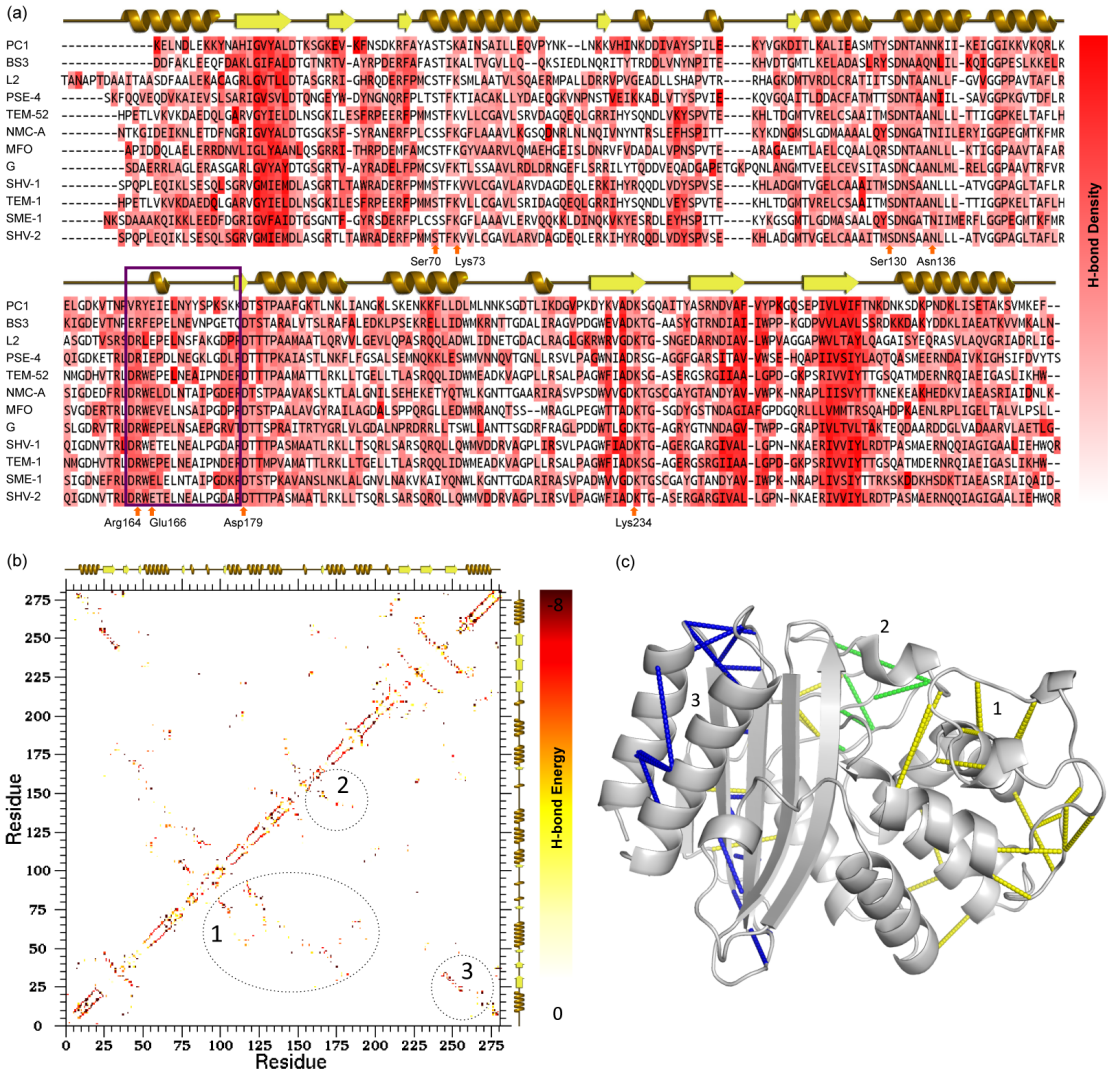
Conservation in H-bond networks. (a) H-bond density is plotted per residue, which identifies regions rich in H-bond interactions. The Ω-loop is shown inside purple box and active site locations are marked as well. (b) Overlapped H-bond contact maps reveal three important sites important for maintaining the active site structure integrity and substrate catalysis. (c) The CA-CA atoms of residues at the corresponding sites (1, 2 and 3) are depicted by yellow, green and blue lines respectively. For better visualization only strong H-bond connections have been displayed on the structure.

We employ a simple but effective approach for comparing H-bond networks by plotting the H-bond density per residue and the H-bond contact maps to visualize essential differences (Figure 7). There is a rich density of H-bonds at strand β1, the Ω-loop and β9, which are conserved throughout the family (Figure 7a). An overlapped H-bond contact map of all the twelve BL structures gives us an insight of regions with strong H-bond interaction, where each pixel is color-coded by H-bond strength (Figure 7b and 7c). The site labeled 1 shows strong interactions between three regions that extend over all key catalytic sites. Similarly, experimental studies [46] have highlighted the importance of strong interactions between (*i.*) Lys-73 and Glu-166, (*ii.*) Arg-164 and Asp-179, and (*iii.*) Asn-136 and Glu-166. In those reports, the authors emphasize that removing any of these interactions can make the enzyme catalytically inefficient, while also disturbing its stability. Site 2 on the contact map highlights a strong interaction network within the Ω-loop region. Based on its location, the network is thus assumed to be important for maintaining functionality. Site 3 illustrates the presence of strong interactions between strands β1 and β9, which is assumed related to structural stability. Furthermore, strong H-bond interactions are observed at secondary structures as expected. Another interesting observation is that sites 1 and 3 represent the two distinct BL domains (as defined by SCOP), whereas site 2 is at the interface between the two domains and overlaps the active site, thus further stressing the importance of the Ω-loop region.

The conserved regions of high H-bond density, which is most pronounced in secondary structure elements and the Ω-loop region, leads to conservation of backbone rigidity. However, local H-bond conservation does not necessarily indicate that their energies are equivalent, which could lead to the observed differences within backbone flexibility and cooperativity correlation. As such, we also compare the H-bond contact maps to the observed property differences to the evolutionary and antibiotic specificity patterns. As with most of the other physiochemical quantities, differences within the H-bond networks again reflect the evolutionary, but not antibiotic specificity patterns. In fact, the relationship between the H-bond contact map and the evolutionary patterns is the strongest relationship (lowest p-value) observed. A statistically significant relationship is also observed by looking at the H-bond density of each residue.

### Conclusions

The BL enzyme family represents an interesting case study in protein family evolution. While conservation of function is the primary driving force in the evolution of most protein families, rampant antibiotic overuse has introduced new pressures leading to new resistance activities that reflect subtle differences within substrate specificity. The bulk of these changes are trivially explained by steric changes within the BL active site [52]; however, it has never been determined if antibiotic specificities are related to global physiochemical properties. We clearly demonstrate that they are not. On the other hand, all of the global properties considered here vary in a systematic way that reflects the family's phylogeny. Physiochemical properties diverged early in the evolution of the family, leading to outgroups with conserved properties therein, and systematic differences between them. Related, stratifying the dataset based on the environmental condition the bacteria they are from also parallels the variations within the global physiochemical properties. Interestingly, differences within local properties at the Ω-loop region do not reflect either because variation is suppressed based on functional requirements.

The differences and similarities within two pairs of class-A BLs encapsulate our results. First, consider the PC1 and G pair of penicillinases. The phylogeny clearly indicates that these proteins diverged early in the evolutionary history of the family, yet they have identical antibiotic specificities. In spite of the functional conservation, the evolutionary divergence has led to very different global physiochemical properties, which can be seen most starkly in the global electrostatic potential maps (Figure 3) and cooperativity correlation plots (Figure 6). Conversely, MFO is from the same evolutionary outgroup as G, but they have vastly different specificities (MFO has extended spectrum activities and can be classified as a cephalosporinase that can also hydrolyze monobactams). Despite substrate specificity differences, the electrostatic potential maps and cooperativity correlations plots are very similar as a consequence of their close evolutionary relationship. This point is particularly noteworthy and cautionary because it suggests that new antibiotic specificities, including extended spectrum activities, can emerge from the background of nearly any set of electrostatic and dynamic properties through local changes that do not significantly alter the global properties.

## Methods

### Continuum electrostatic calculations

Additions of hydrogen atoms, residue p*K_a_* calculations and intramolecular electrostatic interactions have been performed on energy minimized protein structures using H++ web server [53]. Hydrogen atoms were added and their positions optimized (MD based) after calculating ionization states of the titratable residues using Poisson-Boltzmann continuum electrostatics. The server uses MEAD suite of programs, and detailed information of the algorithm can be found here [53]. The salinity and pH conditions are kept consistent with the conditions used in the original DSC experiment, i.e., 0.06M salt concentrations and pH 7.0; and a solvent dielectric constant of 80 and an interior protein dielectric of 6. Residue acidity and basicity changes (Figure 2a) are calculated with respect to model p*K_a_* values from [54]. The .pqr file generated from H++ containing charge and radii information is fed into APBS [55] to generate electrostatic potential data. The protein is centered on a 65×97×65 grid. The electrostatic potential maps in Figure 3 are displayed at +/−1.0 kcal/mol.

### The distance constraint model

The DCM is fundamentally based on a free energy decomposition scheme that explicitly accounts for nonadditivity within entropic components [56]. Therein, macromolecular structure is described as an ensemble of network rigidity topological frameworks, where intramolecular interactions are modeled by distance constraints and vertices represent atomic positions. Interactions such as covalent bonds, hydrogen bonds, and local residue conformational states are modeled as a three-dimensional network (or framework) of distance constraints. Distance constraints restrict the amount of available degrees of freedom (DOF) between adjacent vertices, and each framework is used to describe a set of similar geometric conformations that share a common set of interactions. Distance constraints are associated with a component enthalpy and entropy, and the total enthalpy of a given framework is simply the sum over the set of distance constraints. The free energy of a given framework is calculated by:
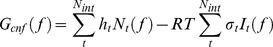
(1)where *N_int_* is the number of different types of modeled interactions, *h_t_* and σ*_t_* define enthalpy and entropy of a single distance constraint used to model interaction type *t*. *N_t_* is the number of times interaction *t* occurs in a given framework, 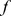, and *I_t_* is the number of independent constraints of type *t*, where, *I_t_* is always less than or equal to *N_t_*.

However, entropy components are nonadditive due to correlations within the dynamics, thus simple sums result in drastic overestimations of the total entropy. Entropy components are additive only over the set of independent DOF [57], [58]. The DCM employs efficient network rigidity graph algorithms [59]–[61] to quickly differentiate the independent and redundant constraints. Adding a constraint within a flexible region of the network removes a single DOF, whereas adding a constraint to a rigid region has no entropy affect because all DOF in that region have already been consumed. The network rigidity algorithm recursively adds distance constraints based on their order of entropy (from smallest to largest), rigorously providing the lowest upper bound estimate of the total entropy [62]. Note that a given chemical interaction can be modeled by more than one constraint. For example, torsion force is modeled as one constraint, H-bonds and covalent bonds as five. The free energy of a given protein would simply be based upon the above calculation if thermal fluctuations did not occur. Hence, topological differences arise due to fluctuating interactions, which account for the forming and breaking of weak interactions at equilibrium. Covalent bonds are quenched, meaning they need not be parameterized since the set is uniform across the ensemble. In the mDCM, torsion angle forces are segregated into native and disordered states, and H-bonds can be present or not. Salt bridges are modeled as a special case of H-bonds. For BL, the number of microstates is astronomical (∼2^1850^); as such, the process of solving the mDCM for proteins is based on heterogeneous mean field theory [62]. A free energy landscape is defined by order parameters that specify the number of H-bonds (*N_hb_*) and native torsions (*N_nat_*) within a given macrostate. The free energy of a given macrostate is given by the free energy functional:
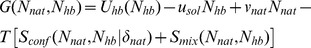
(2)where *v_nat_* and δ*_nat_* correspond to the enthalpy and entropy associated with a native torsion. The corresponding values of *v_dis_* and δ*_dis_* have been fixed in prior works [63]. The total H-bond energy, *U_hb_*, is determined using a modified [50] empirical potential [64], which the component entropy is linearly related to. When a H-bond breaks, there is an enthalpically compensating interaction with solvent that is described by *u_sol_*. While not explicitly specified in Eq. 1, the total conformational entropy, *S_conf_*, is appropriately attenuated by the probability of a distance constraint to be independent to account for nonadditivity. The probability for a distance constraint to be independent is determined by Monte Carlo sampling of topological frameworks that satisfy the order parameters. The mixing entropy term, *S_mix_*, arises from the various combinations that can satisfy the order parameters. The hydrophobic interactions are indirectly included in the *u_sol_* and *v_nat_* parameters as discussed in [37], [65], i.e., H-bond formation implicitly accounts for the hydrophobic contacts.

Critical to the work presented here, the mDCM provides a large number of mechanical descriptions of structure referred to as Quantitative Stability/Flexibility Relationship (QSFR). Flexibility implies conformational diversity, whereas rigid regions are structurally conserved. The mechanical origins of flexibility and rigidity are directly linked to conformational entropy. Hence, these thermodynamic and mechanical quantities combine to define QSFR. To be precise, the free energy of a protein can be expressed as a function of global flexibility *θ*, where *θ* is equal to the average number of independent degrees of freedom divided by the total number of residues. As *θ* increases, the protein transitions from a folded state to an unfolded state. The ensemble averaged mechanistic or QSFR quantities of a protein are calculated using conformations in the native basin of the protein.

Common QSFR metrics include the flexibility index (FI) and cooperativity correlation (CC). FI is a local description of backbone dynamics. Positive FI values quantify the number of excess DOF within a region, whereas negative values quantify the number of redundant constraints (Figure 4b). A region is said to be isostatically rigid (meaning marginally rigid) when FI = 0. As described above, CC plots identify all pairwise residue-to-residue couplings across the structure. In both metrics, the presented values represent the Boltzmann-weighted average across the native structure free energy basin.

### Model parameterization

**Figure 8:**
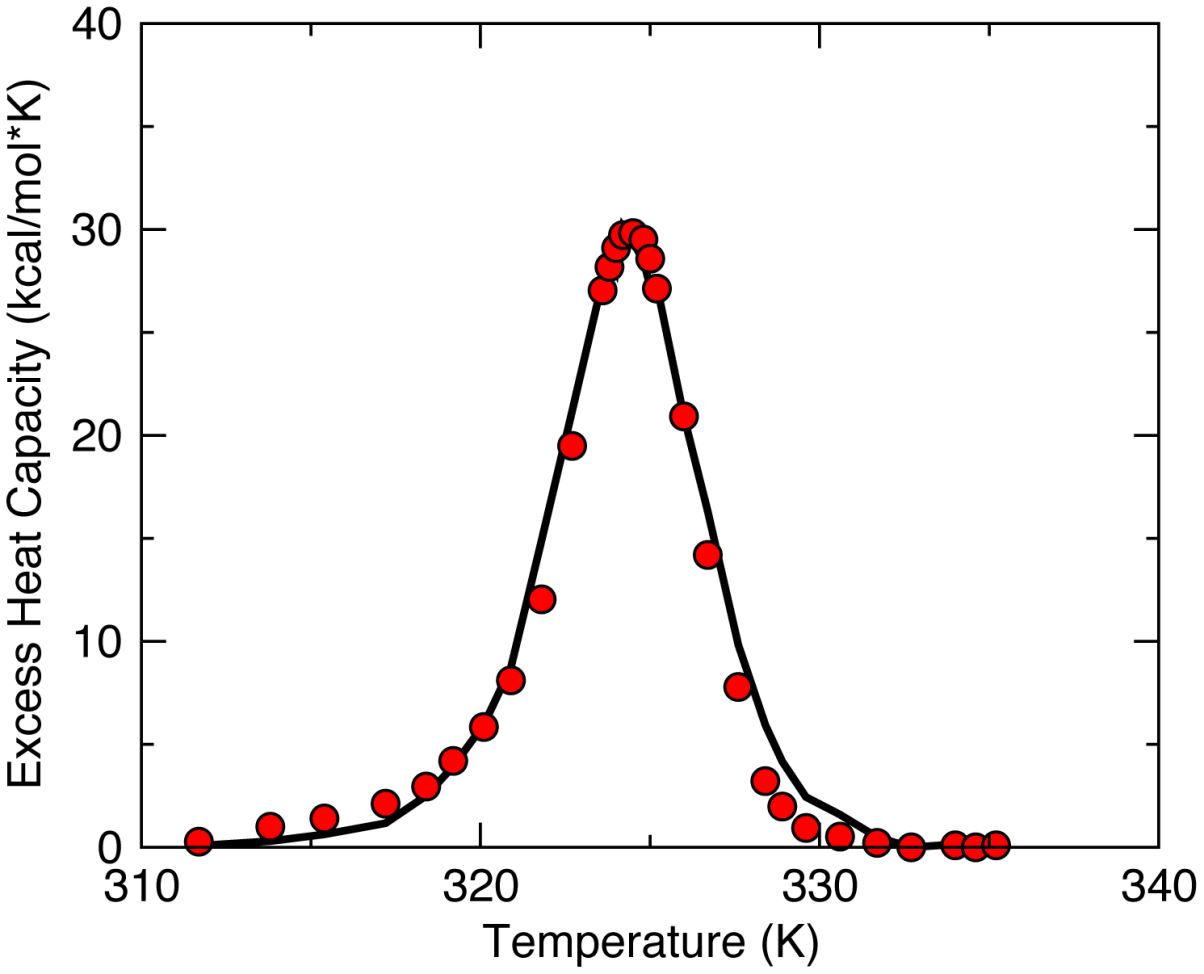
The best-fit heat capacity curve by mDCM is shown with *u_sol_* = −2.61, *v_nat_* = −0.32 and δ*_nat_* = 1.61, which are within normal ranges established by our previous studies (solid line = model and symbols = experiment). The three fitting parameters are required to calculate free energy of the protein accurately using Eq. 1.

The mDCM is parameterized by finding values of (*u_sol_*, *v_nat_*, δ*_nat_*) that best reproduces the experimental *C_p_* data using simulated annealing method (Figure 8). We parameterize the model using the *C_p_* curve from *B. cereus* [66] and the evolutionarily closest structure BS3. Focusing on our group of twelve class-A BL proteins with well-conserved structures of the same function, we have transferred the three adjustable parameters obtained from above to all the other members, which is an approach we have used previously [39], [50]. With this fixed parameterization, we have confirmed that mDCM correctly predicts all BL orthologs to have a single peak in *C_p_* and a two state folding/unfolding transition in free energy. Apart from these twelve BLs, an attempt was made to calculate QSFR quantities on three other BL structures (1IYS, 1HZO, and 1E25), but their free energy landscapes were not two-state, so they were excluded. This is not to say that having a continuous transition is necessarily wrong; the model has been shown to not give two-state behavior when its inappropriate (i.e., met-myoglobin) [63]. Nevertheless, in the absence of external biophysical characterizations, which to the best of our knowledge do not exist, it is impossible to know if the atypical behavior is real or simply an artifact of pushing the parameterization too far, thus they were excluded.

We have consistently demonstrated that while thermodynamic quantities (i.e., *T_m_*) are somewhat sensitive to parameterization and input structure resolution, the mechanical FI and CC quantities are mostly robust to parameter differences. Nevertheless, a single parameter set across the dataset, guarantees that QSFR differences only arise from structural differences. Also, results from our previous works [37], [39]–[41] have demonstrated that QSFR properties are insensitive to parameterization, and have minimal influence on CC and FI values. As such, the conclusions regarding changes in QSFR properties are robust.

### Dataset preparation

**Figure 9:**
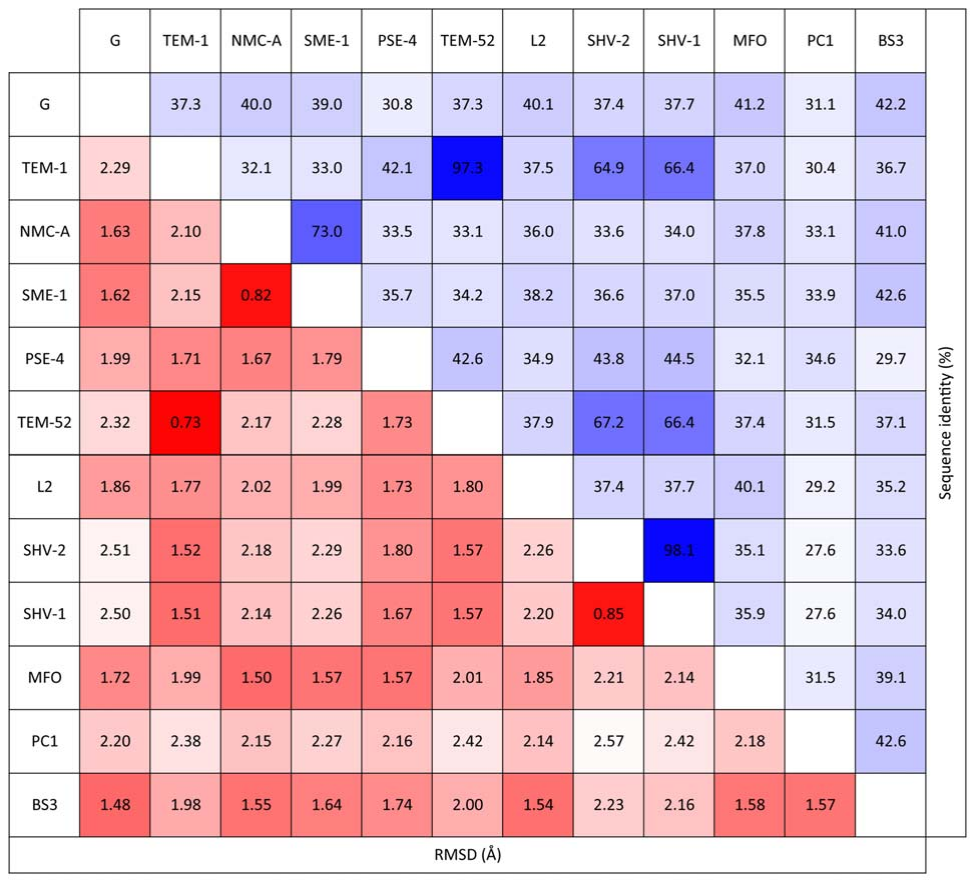
Dataset similarity. All-to-all percent sequence identity (blue) and structural RMSD (red, in units of Å) are provided to highlight (dis)similarity.

In this study, twelve different class-A BL structures are investigated to provide a large evolutionary cross-section for detailed analysis [35], [67]–[77], while maintaining a feasible number for data and visual assessment. Our dataset is based on a set of high-resolution BL structures without any internal missing residues. The resolution and R-values of all structures are respectively less than or equal to 2.4 Å and 0.22. As provided in Table 4, three out of twelve structures exhibit penicillinase activity while the rest belong to one of the following classes: broad-spectrum, extended-spectrum, carbapenamase, cephalosporinase or carbenicillinase. Moreover, all enzymes are inhibited by clavulanic acid and their structures are remarkably similar; the pairwise α-carbon root mean square deviation (RMSD) ranges from 0.73 to 2.57 Å (cf. Figure 9).

**Table 4:** Structural and catalytic characterization of the dataset.

Organism	Enzyme Name	PDBID	Res (Å)	R-value	Function Class^1^	Extended spectrum?	Substrate
*S. albus*	G	1BSG	1.9	0.15	2a	No	Penicillins
*E. coli*	TEM−1	1BTL	1.8	0.16	2b	Yes	Penicillins & early cephalosporins
*E. cloacae*	NMC-A	1BUE	1.6	0.19	2f	Yes	Carbapenems
*S. marcescens*	SME-1	1DY6	2.1	0.18	2f	Yes	Carbapenems
*P. aeruginosa*	PSE-4	1G68	2.0	0.17	2c	No	Carbenicillin
*K. pneumonia*	TEM-52	1HTZ	2.4	0.22	2be	Yes	Extended-spectrum cephalosporins & monobactams
*S. maltophilia*	L2	1N4O	1.9	0.16	2e	Yes	Extended-spectrum cephalosporins
*K. pneumoniae*	SHV-2	1N9B	0.9	0.13	2be	Yes	Extended-spectrum cephalosporins & monobactams
*K. pneumoniae*	SHV-1	1SHV	2.0	0.18	2b	Yes	Penicillins & early cephalosporins
*M. foruitum*	MFO	2CC1	2.1	0.17	2be	Yes	Extended-spectrum cephalosporins & monobactams
*S. aureus*	PC1	3BLM	2.0	0.16	2a	No	Penicillins
*B. licheniformis*	BS3	4BLM	2.0	0.16	2b	Yes	Penicillins & early cephalosporins

1 Functional class is defined by Bush et al. [17], [84].

### Phylogeny

For expanding sequential coverage, we collect approximately 1100 sequences after searching through the nonredundant protein database using BLASTP [78]. The protein sequence culling algorithm PISCES [79] is employed to filter sequences at 98% mutual sequence identity cutoff. This reduced dataset, which also includes twelve class-A BL protein sequences, is further aligned by MUSCLE [80] followed by phylogenetic tree construction using maximum-likelihood, meaning the phylogenetic tree shown in Figures 3 and 6 is purely derived from sequence information. The twelve BL protein sequences span across the evolutionary tree, which provide a robust structural coverage as well. However, we arrange these twelve BL sequences independent of the larger set, using both sequence and structural information by Protein Align tool in MOE [81], to achieve better visual comparison across our set.

### Hydrogen bond network

H-bond density for a residue *i* is defined as:
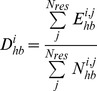
(3)where 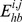 is the hydrogen bond energy between residue *i* and *j*, and 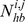 is the number of hydrogen bonds formed between residue *i* and *j*. The summation of energies divided by total number of hydrogen bonds provides hydrogen bond density at per residue level (Figure 7a). The hydrogen bond network contact map, shown in Figure 7b, is an overlapped network of all twelve BL proteins. The residue positions on the network follow multiple sequence alignment as described above. As such, identical donor and acceptor residue pair positions across the dataset are achieved for easy visual network assessment of hydrogen bond energies.

### Relating physiochemical and clustering patterns

To determine the statistical significance of our results, we have developed a cluster matching score, *S*. In the case of the evolutionary patterns, the clusters are defined by the various colors in the phylogeny (cf. Figure 3), which were determined using a constant cut-level through the rectangular dendrogram. The matching score is calculated using the following equation:
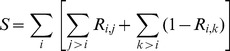
(4)where *R_x,y_* is the correlation between a vector of physiochemical properties associated with protein *x* and *y*. The equation has been developed to evaluate both intra-cluster similarities (all pairs *i,j*) and inter-cluster variability (all pairs *i,k*). That is, random data would not provide a good score because the intra-cluster on the left would be negligible, whereas the inter-cluster term on the right would be negligible if the data were well conserved throughout the family. Conversely, a perfect score, *S* = 66, would occur when all *R_i,j_* = 1 and *R_i,k_* = 0, which is defined by the number of protein pairs in the dataset. For example, in the case of FI, the vectors are defined by all values of FI for residues present across the 12 structures, meaning alignment positions with gaps are ignored. The same is done for the residue H-bond density quantity. Residue charge and residue p*K_a_* shifts are very similar, but the only difference is the vector length is only for the subset of titratable residues. In the case of the *N*x*N* H-bond network and CC plots, the vector length is *N*(*N*-1)/2. The electrostatic network is also *N*×*N*; however, *N* is a relatively small number based on the residues identified within the active site electrostatic network. Because the size of these vectors is small, we also wanted to consider an alternate Rand Measure (RM) [82] that only considers set identity, which we have with good results in alternate work [83]. In this case, the *R_x,y_* correlations are simply replaced with RM, which also scales between zero and one, and gives nearly identical results.

Statistical significance of the match scores is determined by comparing the real calculated score, *S_real_*, to an ensemble of random values, *S_shuffle_*, where the cluster identities have been randomly shuffled. We perform a z-test on each of the property comparing *S_real_* to the *S_shuffle_* distribution. The corresponding p-values are provided in Table 3. Statistical significance is assumed if the p-value is less than 0.05, meaning it is highly unlikely to obtain *S_real_* from the randomized distribution. This indicates that the matching between the physiochemical properties (i.e., intra-cluster conserved properties and systematic inter-cluster differences) and evolutionary groups is statistically significant and represents true sequence/property relationships. Relationships with environmental condition and antibiotic specificity patterns are calculated in the exact same way. The clusters for the former are defined above, whereas the antibiotic specificity clusters are use the Bush and Jacoby classification scheme [17], [84] (cf. Table 4).
